# A versatile system for rapid multiplex genome-edited CAR T cell generation

**DOI:** 10.18632/oncotarget.15218

**Published:** 2017-02-09

**Authors:** Jiangtao Ren, Xuhua Zhang, Xiaojun Liu, Chongyun Fang, Shuguang Jiang, Carl H. June, Yangbing Zhao

**Affiliations:** ^1^ Center for Cellular Immunotherapies, Perelman School of Medicine, University of Pennsylvania, Philadelphia, PA, USA; ^2^ Department of Pathology and Laboratory Medicine, Perelman School of Medicine, University of Pennsylvania, Philadelphia, PA, USA

**Keywords:** CRISPR/CAS9, T lymphocytes, chimeric antigen receptors, PD-1, CD95

## Abstract

The therapeutic potential of CRISPR system has already been demonstrated in many instances and begun to overlap with the rapidly expanding field of cancer immunotherapy, especially on the production of genetically modified T cell receptor or chimeric antigen receptor (CAR) T cells. Efficient genomic disruption of multiple gene loci to generate universal donor cells, as well as potent effector T cells resistant to multiple inhibitory pathways such as PD-1 and CTLA4 is an attractive strategy for cell therapy. In this study, we accomplished rapid and efficient multiplex genomic editing, and re-directing T cells with antigen specific CAR via a one-shot CRISPR protocol by incorporation of multiple gRNAs in a CAR lentiviral vector. High efficient double knockout of endogenous TCR and HLA class I could be easily achieved to generate allogeneic universal CAR T cells. We also generated Fas-resistant universal CAR T cells by triple gene disruption. Simultaneous gene editing of four gene loci using the one-shot CRISPR protocol to generate allogeneic universal T cells deficient of both PD1 and CTLA-4 was also attempted.

## INTRODUCTION

Engineered T cell receptor (TCR) and chimeric antigen receptor (CAR) T cell treatment of cancer patients has shown promising results [1–6]. Most of these successes were observed in clinical trials during the treatment of B cell malignancies. Solid tumors are considerably more difficult to eliminate due to the complex inhibitory factors in the tumor microenvironment. Immune escape mediated by inhibitory pathways via the interaction of activated killer T cell receptors with their ligands, such as programmed cell death1 (PD-1) [7, 8], and cytotoxic T-lymphocyte antigen 4 (CTLA-4) on T cells [9] is another major factor. In addition, the function of T cells is always impaired by exhaustion, particularly in patients with chronic infections and cancers, during which the T cells are exposed to persistent antigen and/or inflammatory signals. Exhausted T cells lose their robust effector functions, express multiple inhibitory receptors and are defined by an altered transcriptional program. PD-1, CTLA-4, domain-containing protein-3 (TIM-3)[10–12], and lymphocyte-activated gene-3 (LAG-3)[13] have been reported to play significant roles in T cell exhaustion. Reversing T cell exhaustion by blocking the PD-1 or CTLA-4 checkpoint has shown promising clinical results. Thus, the generation of T cells resistant to multiple inhibitory pathways is expected to improve the function of CAR T cell therapy for solid tumors.

Current TCR and CAR clinical trials utilize autologous T cells and might thus be hampered by the poor quality and quantity of T cells as well as by the time and expense necessary to manufacture autologous T cell products. These limitations could potentially be circumvented by the use of allogeneic T cells. However, the endogenous TCR on allogeneic T cells may recognize the allo-antigens of the recipient, leading to graft-versus-host disease (GVHD). Furthermore, the expression of HLA on the surface of allogeneic T cells causes rapid rejection by the host immune system. Therefore, simple and efficient methods are needed for the multiplex genomic editing of T cells [14, 15].

The Fas receptor (also known as CD95 and APO- 1) is a member of the tumor necrosis factor α family of death receptors that mediate T-cell responses [16–18]. Fas/FasL-induced cell death is also involved in T cell apoptosis and thus affects the outcome of immunotherapy [19–21]. Reports indicate that CAR T cell activity is attenuated due to cell Fas-FasL-dependent activation induced cell death (AICD) [22]. Thus, ablating Fas-induced cell death using a genetic approach might lead to an enhancement of CAR T cell function.

The CRISPR/Cas9 system has recently emerged as a potentially robust alternative for inducing targeted genetic alterations and as a process for multiplex genomic engineering [23–25]. Current T cell gene editing methods are unable to achieve highly efficient multiplex gene editing in human primary T cells due to low gene editing efficiency or high transfection-associated toxicity. In the present study, we developed a rapid and efficient one-shot CRISPR protocol for multiplex genome editing by incorporating multiple gRNA expression cassettes in a single CAR lentiviral vector to generate genetically edited and modified CAR T cells. We generated allogeneic universal CAR T cells via the double knockout of endogenous TCR and HLA class I (HLA-I). We also generated Fas-resistant universal CAR T cells by triple gene disruption and PD1 and CTLA-4 dual inhibitory pathway-resistant universal CAR T cells by quadruple gene disruption. We also demonstrated the feasibility of generating precise genetically modified CAR T cells by high-fidelity Cas9s with the one-shot platform.

## RESULTS

### Efficient generation of gene ablated CAR T cells with the one-shot CRISPR system

**Figure 1:**
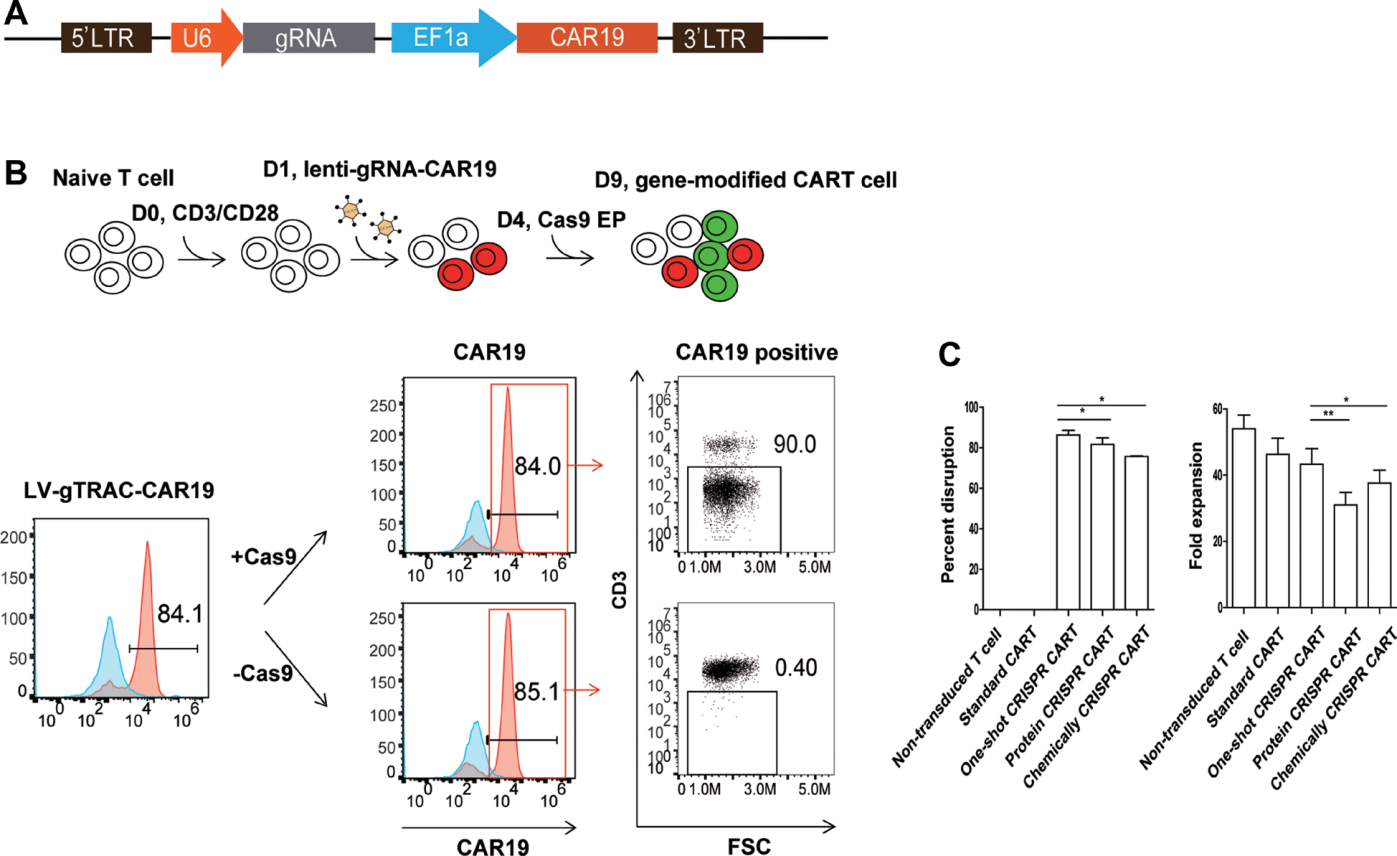
Efficient TCR disruption in T cells with the one-shot CRISPR system (**A**) Schematic design of the one-shot CRISPR constructs. (**B**) Generating TCR/CD3 disrupted CAR T cells with the one-shot CRISPR system. As demonstrated by the flow chart, T cells were first activated by CD3/CD28 beads for one day and then transduced with lentiviral CD19-CAR-TRAC-gRNA. T cells were electroporated with Cas9 mRNA on day 4 after stimulation. TCR/CD3 disruption was measured by flow cytometry on day 8 (*n* = 3). (**C**) Relative fold proliferation of T cells with or without CRISPR editing after the standard CD3/CD28 beads expansion cycle (*n* = 3). gGene, Gene-gRNA. EP, electroporation. *P < 0.05, **P < 0.01, by the Mann-Whitney test.

**Figure 2:**
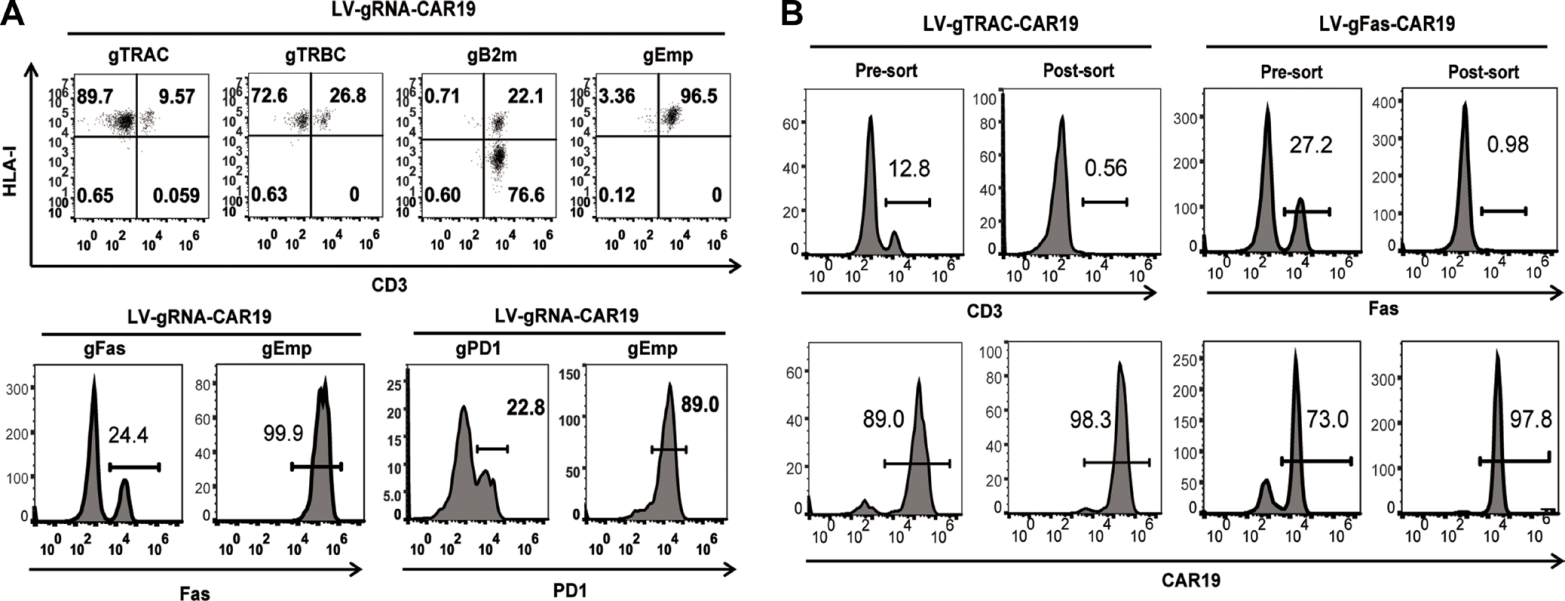
Efficient gene ablation with the one-shot CRISPR system (**A**) Measurement of specific gene disruption with the one-shot CRISPR. gRNAs were designed targeting either TRAC, TRBC, B2m, Fas or PD1, an empty gRNA was used as a control. PD1 ablation was confirmed and measured on day 3 after the re-stimulation of CD3/CD28 beads. Gene disruption was confirmed by flow cytometry. gEmp:Empty-gRNA. (**B**) Enrichment of CD3 or Fas genetically edited CAR T cells by negative selection. Targeted gene and CAR expression before and after selection was measured by flow cytometry.

Because gRNA is more prone to degradation than the Cas9 protein or mRNA, which is a major limitation for highly efficient gene editing, we proposed that efficient gene editing can be achieved by the constitutive expression of gRNA. To test this hypothesis, we expressed a gRNA targeting TCR α chain constant region (TRAC) under a U6 promoter with a CD19 CAR (CAR19) driven by an EF1α promoter in a lentiviral vector (Figure 1A). Both a CAR and TRAC-gRNA can be expressed after transduction into human primary T cells. As determined by flow cytometry and quantitive PCR, CAR19 and gRNA expression reached peak and stayed stable 3 days post transduction (Supplementary Figure 1A, 1B). A single electroporation of Cas9 mRNA (one-shot CRISPR) 3 days after transduction was conducted, over 90% (87 ± 4.3%) of the CAR-positive cells lost CD3 expression (Figure 1B) compared with Cas9 protein and gRNA (protein CRISPR) co-delivery, which yielded 82% (81.7 ± 5.7%, *P* = 0.0433) CD3 disruption, and Cas9 mRNA and chemically modified gRNA (chemical CRISPR) co-delivery, which yielded 76% (75.7 ± 0.61%, *P* = 0.0276) CD3 disruption. The cells could be expanded over 45-fold (43.3 ± 8.1%) using a standard CAR T cell expansion process compared with 36-fold (31 ± 6.6%, *P* = 0.0068) and 39-fold (37.7 ± 6.7%, *P* = 0.0117) for protein CRISPR and chemical CRISPR, respectively (Figure 1C). Efficient gene ablation was also achieved by targeting the TCR β chain constant region (TRBC), Fas and PD1. Beta-2 microglobulin (B2m) is an essential subunit of the HLA-I molecule, and the disruption of B2m produced highly efficient HLA-I ablation from the T cell surface (Figure 2A). The CD3-negative (CD3^neg^) and Fas-negative (Fas^neg^) cell population could be enriched by negative selection. Enrichment of genetically edited cells also enriched T cells that expressed CAR because only cells expressing a CAR expressed gRNA. (Figure 2B). DNA was extracted from enriched TCR/CD3^neg^ CAR^+^ T cells to determine the frequency of TRAC disruption. 89.3% gene disruption was observed by T7E1 assay (Supplementary Figure 2A). Thus, by utilizing the one-shot CRISPR system, we could rapidly and efficiently generate genetically edited CAR T cells, and a pure population of CAR T cells could be obtained by enriching the genetically modified cells.

To test whether CRISPR/Cas9 gene editing would affect the effector function of the T cells, the anti-tumor activity was tested by challenging the TCR/CD3^neg^ CAR19 T cells with CD19^+^ Nalm6 leukemia cells. No difference was observed between wild type and TCR/CD3^neg^ CAR19 T cells in terms of killing activity and cytokine secretion (Supplementary Figure 2B, 2C). The results indicate that CRISPR/Cas9 editing of the endogenous TCR does not adversely affect the function of primary T cells for adoptive immunotherapy.

### Double gene ablation to generate universal CAR T cells with the one-shot CRISPR system

**Figure 3:**
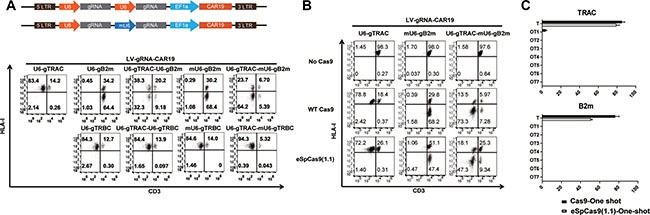
High-fidelity multiple gene ablation by the one-shot CRISPR to generate universal CAR T cells (**A**) TCR and HLA-I double ablation to generate universal CAR T cells. gRNAs were expressed in the one-shot system either under human U6, mouse U6 promoter or a combination of the two. TCR and HLA-I ablation were measured by flow cytometry. mU6, mouse U6 promoter. (**B**) Efficient high-fidelity Cas9 gene editing with the one-shot CRISPR. One-shot CAR T cells were electroporated with either wild-type or high-fidelity Cas9: eSpCas9(1.1) TCR and HLA-I expression were measured by flow cytometry. (**C**) Off-target events of one-shot targeting TRAC and B2m measured by TIDE software; scores below 1.5 are set as 0 (*n* = 3).

As we previously reported, CD3 and HLA-I ablation is essential for abolishing TCR-associated GVHD and HLA-mediated rejection to generate universal CAR T cells [26]. To test the possibility of double gene ablation using the one-shot CRISPR system, we expressed gRNAs that targeted TRAC and B2m in tandem under a U6 promoter. Although highly efficient CD3 or B2m ablation could be achieved by separate targeting, only approximately 30% double gene ablation was observed when both genes were targeted simultaneously. Because a tandem repeat of U6 may cause the recombination of the lentivirus and the gRNA repeats might compete for the U6 RNA polymerase, a murine U6 (mU6) promoter was used for one of the gRNAs instead of a human U6 promoter. Interestingly we found CAR expression was not affected by tandem human U6 promoter (Supplementary Figure 3A), however TRAC and B2m gRNAs expressed under tandem U6 promoter showed reduced expression than TRAC and B2m gRNAs expressed under human U6 and mouse U6 promoter respectively (Supplementary Figure 3B). A population of CD3 and HLA-I double negative CAR T cells greater than 73% (71.3 ± 6.7%) was achieved by the one-shot CRISPR system, and a similar result was observed when enhanced CD3 disruption was achieved by targeting TRAC and TRBC simultaneously (Figure 3A, 3B).

The permanent expression of gRNA might increase the off-target potential of the CRISPR system, so high-fidelity Cas9 mutant eSpCas9(1.1) was tested for more precise gene editing to minimize the off-target potential of the one-shot CRISPR system[27]. Efficient double gene ablation of CD3 and HLA-I was achieved by utilizing eSpCas9(1.1) with the one-shot CRISPR system, yielding over 47% (46.3 ± 2.4%) double negative CAR T cells.

Seven potential off-target sites for either TRAC or B2m were sequenced and measured by TIDE to determine the off-target events produced by the one-shot CRISPR system [28]. We observed very rear off-target events only when targeting TRAC and B2m with Cas9 and no detectable off-target mutagenesis with eSpCas9 (1.1), consistent with our previous finding that CRISPR editing is very precise in T cells [26] (Figure 3C).

### Generation of CAR T cells with triple gene ablation resistant to apoptosis

**Figure 4:**
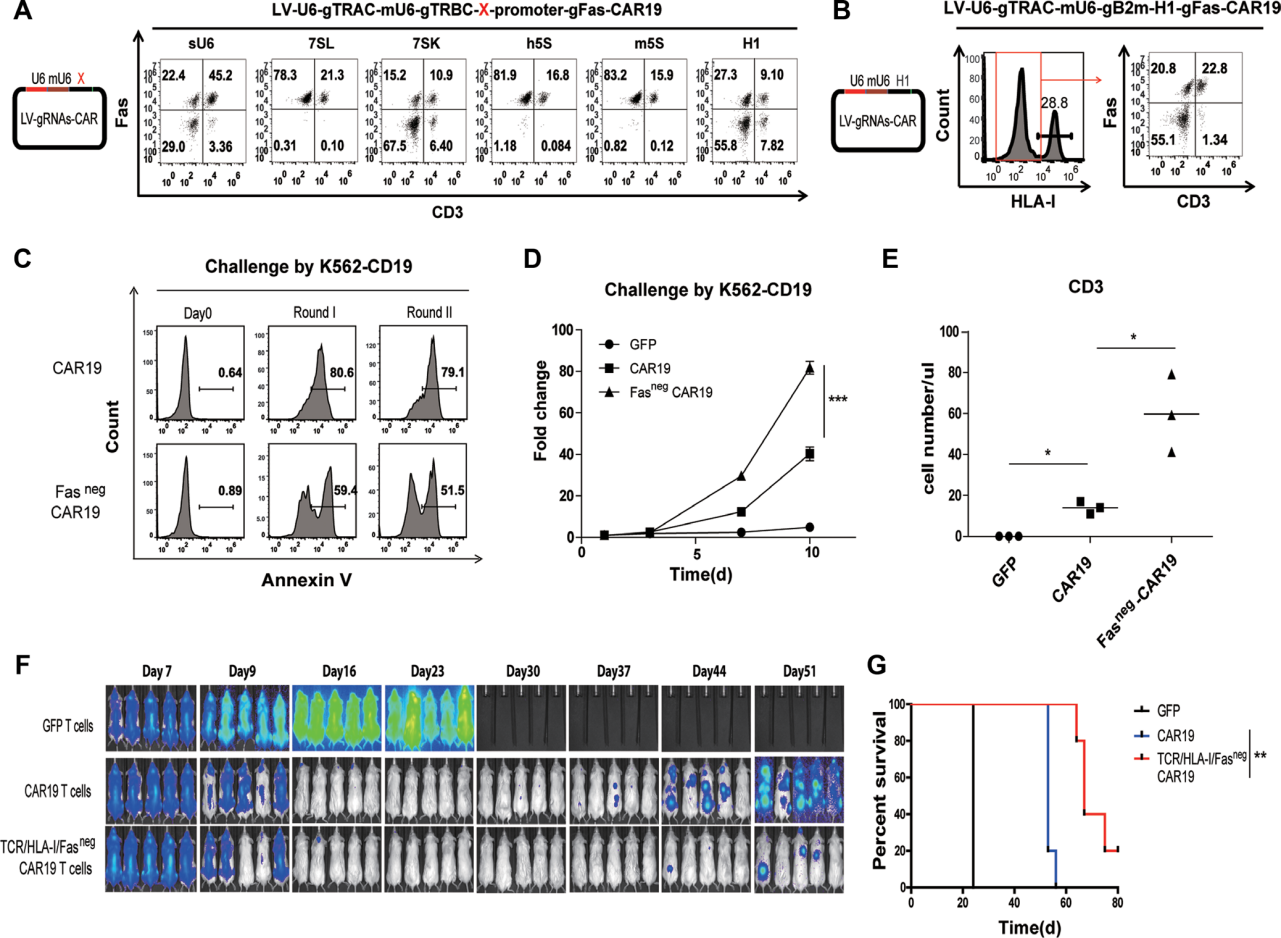
Triple gene ablation with the one-shot CRISPR system to generate universal CAR T cells resistant to apoptosis (**A**) Fas protein reduction by triple knockout using the one-shot CRISPR system with gRNA expressed under different promoters. Triple gene editing using the one-shot CRISPR system was constructed by expressing a TRAC gRNA under a human U6 promoter, a TRBC gRNA under mouse U6 promoter and a Fas gRNA expressed under X promoter. X: sU6, 7SL, 7SK, h5S, m5S, H1. Fas expression was measured by flow cytometry. (**B**) Generation of universal CAR T cells resistant to Fas by triple knockout using the one-shot CRISPR targeting TRAC, B2m and Fas. Protein reduction was confirmed by flow cytometry. (**C**) Reduced AICD of CAR T cells by ablating Fas. Flow cytometry quantification of Annexin V following successive rounds of challenging CAR T cells with K562-CD19 at an E:T ratio of 1:1. (**D**) Increased expansion of CAR T cells stimulated with K562-CD19 at an E:T ratio of 1:1. (*n* = 3). (**E**) Prolonged survival of Fas^neg^ CAR T cells. Tumors were established in NSG mice (*n* = 5 per group) by i.v. injection of 1 × 10^6^ Nalm6 cells. T cells (2 × 10^6^) expressing lentiviral CD19-CAR were infused in a single injection beginning on day 7. T cells expressing lentiviral GFP protein were injected as controls. Peripheral blood from each group was obtained on day 30 and quantified for the presence of CD3 T cells by a FACS Trucount assay. The results are expressed as the mean absolute count per μl of peripheral blood ± SD with *n* = 3 for all groups. The enhanced efficacy of Fas^neg^ CAR T cells was confirmed by (**F**) BLI from each group of mice, and (**G**) overall survival of mice (*n* = 5). ***P < 0.001, by two-way ANOVA plus the Bonferroni post hoc test (b), **P < 0.01, Mann-Whitney test (c) and **P < 0.01, log-rank Mantel-Cox test (h).

To generate CD3, HLA-I and Fas triple negative CAR T cells, Human H1, 7SK, 7SL, 5S, mouse 5S (m5S) and a chimeric U6 (sU6) [29] promoter were tested for the gene disruption efficiency of Fas gRNA in the triple gene ablation lentiviral CAR constructs. Efficient Fas protein ablation was achieved using a human H1 promoter or a 7SK promoter without affecting the gene disruption of CD3 or B2m, and gRNA driven by sU6 showed low gene disruption of Fas, CD3 and B2m. No Fas protein ablation was detected for Fas gRNAs driven by human 5S, 7SL or m5S promoters (Figure 4A). Thus, efficient CD3, HLA-I and Fas triple negative CAR T cells could be obtained with triple knockout using the one-shot CRISPR system with selective promoter combinations (Figure 4B). Robust CAR expression was also detected (Supplementary Figure 4A). To validate the function of one-shot CRISPR triple gene-ablated CAR T cells, the function of the Fas-ablated (Fas^neg^) and CD3, HLA-I, Fas triple-ablated (TCR/HLA-I/Fas^neg^) CAR T cells were tested *in vitro* and *in vivo*. A reduction of apoptosis was observed for the Fas^neg^ CAR19 T cells that were repetitively challenged by K562 that expressed the CD19 antigen (Figure 4C), leading to increased T cell expansion (Figure 4D), which indicated reduced activation induced cell death (AICD) of the Fas^neg^ CAR19 T cells. TCR/HLA-I/Fas^neg^ CAR19 T cells also exhibits elevated degranulation as confirmed by CD107 release, enhanced killing ability and cytokine secretion (Supplementary Figure 4B, 4C, 4D). Reduced AICD was also confirmed by the prolonged survival of the TCR/HLA-I/Fas^neg^ CAR T cells in the peripheral blood of the CAR19 T cell-treated Nalm6-bearing mice (Figure 4E), which in turn enhanced the tumor control efficacy of the CD19 CAR T cells (Figure 4F, 4G).

### Dual inhibitory pathway-resistant CAR T cells generated with the one-shot CRISPR system by quadruple gene ablation

**Figure 5:**
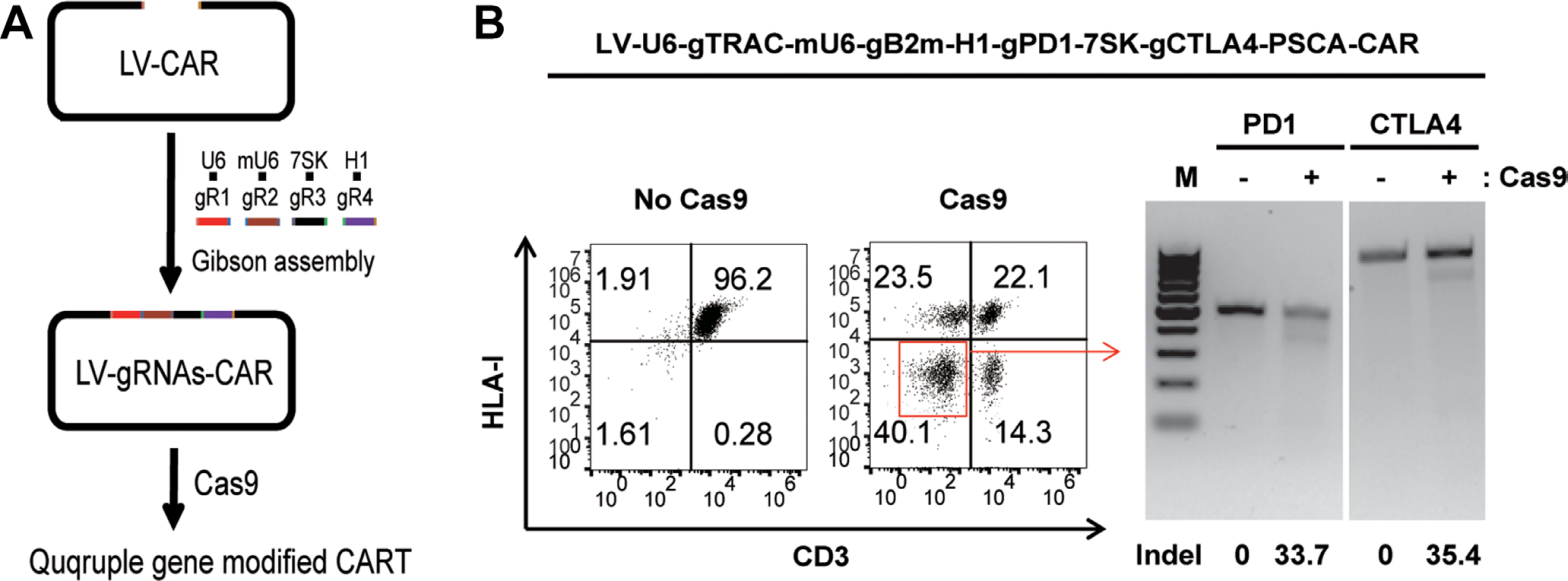
Generating dual inhibitory resistant universal PSCA-CAR T cells by quadruple gene ablation (**A**) A rapid protocol to generate quadruple genetically modified CAR T cells with the one-shot CRISPR system. (**B**) Generation of TCR, HLA-I, PD1and CTLA-4 quadruple gene-disrupted PSCA-CAR T cells. TCR and HLA-I disruption was confirmed by flow cytometry. PD1 and CTLA-4 ablation was confirmed by a T7E1 assay. The percent of gene disruption was calculated and listed with an underline.

Because PD-1 and CTLA-4 have an inhibitory function in T cells, quadruple gene ablation was performed to generate dual inhibitory resistant universal CAR T cells deficient for TCR, HLA-I, PD1 and CTLA-4. A lentiviral vector was constructed for the quadruple gene ablation (Figure 5A). PSCA-CAR expression was determined by flow cytometry (Supplementary Figure 5A). Gene disruption was confirmed by flow cytometry and a T7E1 assay (Figure 5B). The percent of gene disruption decreased as the number of genes manipulated increased due to the competition of the gRNAs for the Cas9 protein. The lower quadruple gene targeting efficiency might have also resulted from less individual gRNA produced by the single lentiviral vector due to the packaging limitation.

## DISCUSSION

Multiplex genome editing is one of most attractive applications of the CRISPR/Cas9 system, and it holds great promise for advancing T cell-based adoptive immunotherapy. In this report, we accomplished highly efficient multiplex genome editing of CART cells with One-shot CRISPR system.

DNA transfection limits the use of multiplex genome engineering in primary T cells due to the substantial toxicity associated with the electroporation of DNA into the T cells [30]. Gene targeting in T cells with lentiviral and adenoviral delivery of CRISPR has low efficiency due to limited transduction rates of Cas9 and gRNA [31, 32]. Kathrin et al. reported efficient PD1 ablation of up to 60% by Cas9 protein together with a single-strand DNA donor [33]. Chemically modified guide RNA has been shown to enhance gene disruption in T cells, but the overall gene targeting efficiency was not comparable to over 90% that was achieved by our one-shot system [34]. Moreover, the low T cell toxicity of the one-shot CRISPR system led to an increased yield of genetically modified CAR T cell products, thus representing a simple and cost-effective scale-up method for clinical application. Double gene knock out in T cells with TALEN to generate universal CAR T cells has been reported; however, the low gene editing efficiency and manipulation resulted in a low product yield as well as a prolonged manufacturing process [35]. Multiplex genome editing using the one-shot CRISPR system facilitates the rapid manufacture of universal CAR T cells deficient in CD3 and HLA-I. An expansion greater than 45-fold can be achieved with a single 9 days standard CAR T cell manufacturing process.

In this report, we also demonstrated the ease with which CAR T cells resistant to apoptosis and inhibition could be achieved with the one-shot CRISPR system. The function of CAR T cells against leukemia can be enhanced by ablation of the Fas receptor. Elevation of AICD resistance and prolonged survival of Fas^neg^ CAR T cells were observed when the CAR T cells were challenged with target tumor cells *in vitro* and *in vivo*.

In this report, we showed that precise gene editing can be achieved with the one-shot system using high-fidelity Cas9s. Researchers in Zhang and Joung's laboratory have improved the specificity of the Cas9 nuclease in two independently discovered Cas9 variants: eSpCas9 and Cas9-HF1 [36, 37]. We observed efficient gene disruption with eSpCas9 (1.1), which indicated the compatibility of the one-shot system with high-fidelity Cas9 variants.

We attempted quadruple gene disruption in T cells with the one-shot system. However, the efficiency decreased as the number of targeted genes increased, which might be a common phenomenon resulting from the competition of the gRNAs for Cas9 when multiple genes are targeted. The packaging size of the lentivirus is also a limitation when more than four genes are manipulated. An improved lentivirus backbone and multiple delivery of Cas9 can be considered for the simultaneous modification of a greater number of genes with the one-shot system.

Because it has the feature of multiplex genome editing, the one-shot system also has great potential for the disruption of genes with various isoforms and family members. In summary, we have demonstrated that the one-shot system is a versatile tool for the rapid and efficient generation of CAR T cells via multiplex genome editing.

## MATERIALS AND METHODS

### Primary human lymphocytes

Primary human CD4 and CD8 T cells were isolated from healthy volunteer donors following leukapheresis by negative selection using RosetteSep kits (Stem Cell Technologies, Vancouver BC, Canada). All specimens were collected under a University Institutional Review Board-approved protocol, and written informed consent was obtained from each donor. Primary lymphocytes were stimulated with anti-CD3/CD28 Dynabeads (Life Technologies, Grand Island, NY). T cells were cryopreserved at day 9 in a solution of 90% fetal calf serum and 10% dimethylsulfoxide (DMSO) at 1 × 10^8^ cells/vial.

### Chimeric antigen receptor T cell generation

Primary lymphocytes were stimulated with anti-CD3/CD28 Dynabeads for 1 day, then transduced with lentivirus encoding CD19-CAR or PSCA-CAR. Cells were split every 2 days and harvested on day 9.

### Generation of one-shot constructs for lentiviral transduction

CD19-CAR was synthesized and subcloned into pTRPE lentiviral vectors, and the one-shot system was constructed based on the lentiviral pTRPE-CD19-CAR by adding gRNA sequence under the control of different promoters.

### CAR and one-shot CAR T cell production

CD4 and CD8 T cells at an equal ratio were stimulated by CD3/CD28 Dynabeads and transduced by lentiviral CD19 (PSCA)-CAR or the one-shot CRISPR CD19 (PSCA)-CAR on day 1. Cells were split every 2 days and harvested on day 9.

### CAR T cell gene editing with one-shot protein and chemical CRISPR

Cas9 mRNA was transcribed *in vitro* using mMESSAGE mMACHINE T7 ULTRA kits (Life Technologies, AM1345, Carlsbad, CA). gRNA were transcribed using a HiScribeTM T7 High Yield RNA Synthesis Kit (NEB). Cas9 protein was purchased from PNA Bio (CP01).

For the one-shot CRISPR, 20 μg of Cas9 mRNA was mixed with one-shot CAR T cells. For chemical CRISPR, 20 μg of Cas9 mRNA and 10 μg of gRNA were mixed with CAR T cells. For protein CRISPR, 5 μg of Cas9 protein in storage buffer (20 mM HEPES pH 7.5, 150 mM KCl, 1 mM DTT, and 10% glycerol) was mixed with gRNA dissolved in nuclease-free water and incubated for 10 min at room temperature before being mixed with CAR T cells.

Electroporation of CRISPR reagents with one-shot CAR or CAR T cells was performed with a BTX830 electroporator.

Briefly, T cells were washed three times with OPTI-MEM and re-suspended in OPTI-MEM (Invitrogen) at a final concentration of 1–3 × 10^8^ cells/ml. Subsequently, 0.1 ml of the cells was mixed with IVT RNA and electroporated in a 2 mm cuvette. Twenty micrograms of Cas9 mRNA was electroporated into the cells using a BTX830 (Harvard Apparatus BTX) at 360 V and 1 ms. Following electroporation, the cells were immediately placed in 2 ml of pre-warmed culture media and cultured in the presence of IL-2 (100 IU/ml) at 37°C and 5% CO_2_.

### Guide RNA design

CRISPR/Cas9 can tolerate 1–5 mismatches between gRNA and target sequence, which can lead to off-target nuclease activity, so before screening,

gRNAs containing more than 13 base pairs of complementary sequences to off-target sites are excluded to minimize off-target events.13 gRNAs targeting the first exon of TRAC are tested, 1 with the highest TCR/CD3 disruption is selected for the later experiment.

The gRNA targeting sequences used in this study were as follows:

TRAC-gRNA: AGAGTCTCTCAGCTGGTACA

TRBC-gRNA: GCAGTATCTGGAGTCATTGA

B2M-gRNA: CGCGAGCACAGCTAAGGCCA

Fas-gRNA: GAGGGTCCAGATGCCCAGCA

PD1-gRNA: GGCCAGGATGGTTCTTAGGT

CTLA-4-gRNA: GCAAAGGTGAGTGAGACTTT

### Flow cytometry

The following monoclonal antibodies and reagents were used with the indicated specificity and appropriate isotype controls. From BD Biosciences (San Jose, CA): APC-conjugated anti-CD3 (555335), PE-anti-CD8 (555635and PE-anti-beta-2 microglobulin (551337), FITC-anti-HLA-I (555552). From Biolegend (San Diego, CA): APC-anti-PD1 (329902), PE-anti-Fas (305607). From Beckman Coulter (Pasadena, CA): PE-anti-Vb13.1 (IM2021U). Data were acquired on a FACS Accuri (BD Biosciences, San Jose, CA) using CellQuest version 3.3 (BD Biosciences, San Jose, CA) and analyzed by FCS Express version 3.00 (De Novo Software, Los Angeles, CA) or FlowJo version 7.6.1 (Tree Star, Inc., Ashland, OR).

### Enrichment genetically edited T cells

Cells washed with Auto MACS buffer were incubated for 30 min with CD3 microbeads (Miltenyi Biotec, 130-050-101, Auburn, CA) at 4°C or anti-PE microbeads (Miltenyi Biotec, 130-048-801) after Fas staining. After being washed twice, the cells were passed through an LD column (Miltenyi Biotec, Auburn, CA), and the flow-through fraction was collected for further use.

### Sanger sequencing

The level of genomic disruption of PD1 and CTLA-4 in T cells was determined by a T7 Surveyor Nuclease assay (NEB). The percent target disruption was quantified by densitometry. The PCR primers used for the amplification of the target locus were as follows:

PD1 forward, 5′-GTAATAAAATGCTCAGCACAG AATA-3′

PD1 reverse, 5′-GAGAAAAATATCACCAGCTCA TCT-3′

CTLA-4 forward, 5′- CCCTTGTACTCCAGGAAA TTCTCCA-3′

CTLA-4 reverse, 5′- ACTTGTGAGCTCATCCTGA AACCCA-3′

TRAC forward, 5′- TCATGTCCTAACCCTGATCC TCTT -3′

TRAC reverse, 5′- TTGGACTTTTCCCAGCTGAC AGA -3′

### Real time PCR

SYBR™ Green master mix was purchased from Thermo Fisher (4309155). Genomic DNA was isolated from sorted CAR^+^ T cells. Primers for detection of gRNA expression are listed below; all the gRNAs were measured using the same reverse primer:

TRAC-gRNA forward, 5′- AGAGTCTCTCAGCT GGTACA-3′

B2m-gRNA forward, 5′- GCAGTATCTGGAGTCA TTGA -3′

gRNA reverse, 5′- AAAAAAGCACCGACTCGG TGCCACT -3′

Gapdh forward, 5′- GCTACACTGAGCACCAGGT GGTCTC -3′

Gapdh reverse, 5′- CCCAGCAGTGAGGGTCTCT CTCTTC -3′

### Mouse xenograft studies

All animal experiment protocols were approved and conducted in accordance with the Institutional Animal Care and Use Committee. Studies were performed as previously described with certain modifications (33, 34). Briefly, for the Nalm6 tumor model, 6- to 10-week-old NSG mice were injected with 1 × 10^6^ Nalm6 tumors cells through the tail vein on day 0. The T cell treatment began on day 7 after the tumor inoculation. T cells were administered at a dose of 2 × 10^6^ cells/mouse (2M).

## SUPPLEMENTARY FIGURES AND TABLES


