# Supplementary material for: A versatile system for rapid multiplex genome-edited CAR T cell generation
Source: Oncotarget. 2017 Feb 9;8(10):17002–11. doi: 10.18632/oncotarget.15218 (PMC5370017; doi:10.18632/oncotarget.15218)
Supplement: Supplementary file 1 [file oncotarget-08-17002-s001.pdf]

A versatile system for rapid multiplex genome-edited CAR T cell generation

Supplementary Materials

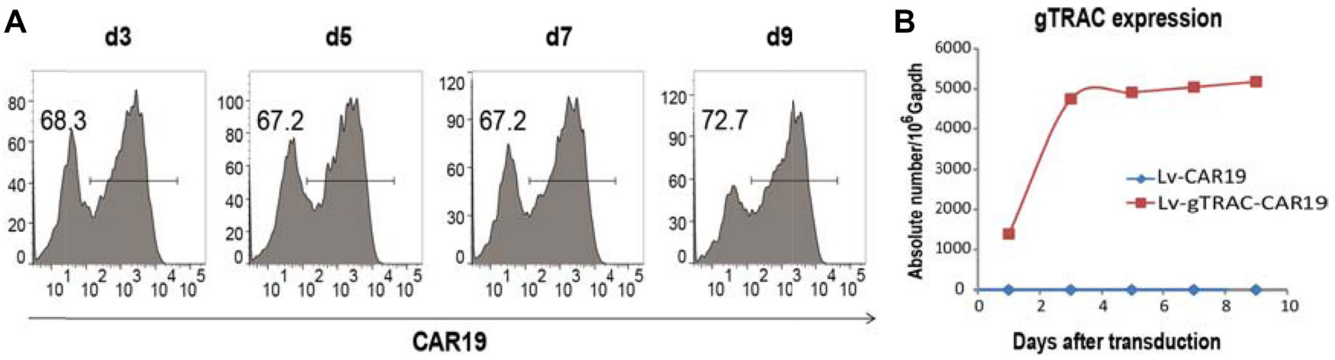

Supplementary Figure 1: Kinetics of one-shot CAR and gRNA expression in T cells. (**A**) Expression of CAR19 in T cells after transduction was measured by flow cytometry. (**B**) Quantitive expression of TRAC-gRNA determined by absolute real-time PCR. Absolute number of gRNA was normalized to 10^6 Gapdh mRNA.

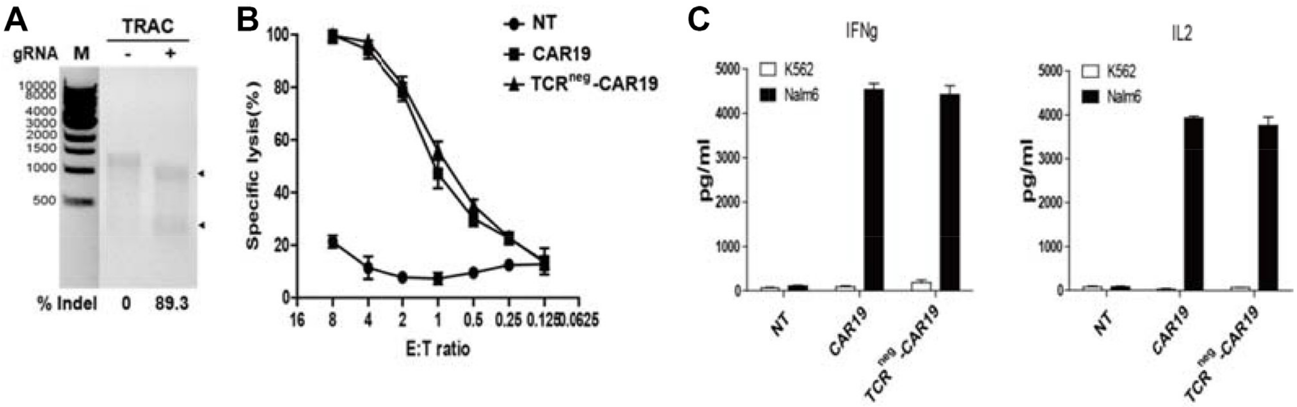

Supplementary Figure 2: CRISPR mediated TCR ablation does not affect CAR19 T cell effector function. (**A**) Amount of TRAC-targeted gene disruption measured by a mismatch-selective T7E1 surveyor nuclease assay on DNA amplified from the cells shown. The calculated amount of targeted gene disruption in TRAC is shown at the bottom. Arrows indicate expected bands. (**B**) Killing ability of wild type and TCR^{neg} CAR19 T cells on Nalm6 target tumor cells. (**C**) Cytokine secretion of wild type and TCR^{neg} CAR19 T cells co-culture with target and non-target tumor cells at an effector to tumor ratio of 1 to 1.

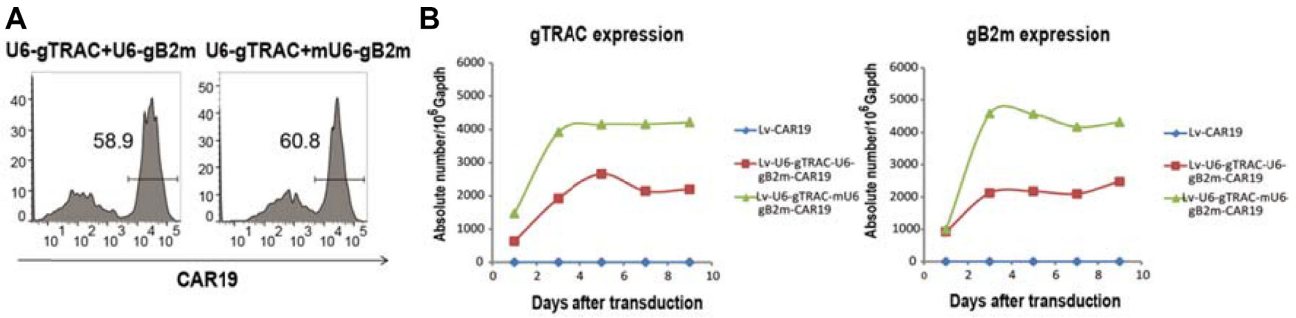

Supplementary Figure 3: Expression of CAR and gRNA in double knockout CAR T cells. (**A**) Expression of CAR19 on T cells re-directed with one-shot Lv-U6-gTRAC-U6-gB2m-CAR19 and Lv-U6-gTRAC-mU6-gB2m-CAR19 lentivirus as determined by flow cytometry. (**B**) Quantitive expression of TRAC and B2m gRNAs in Lv-U6-gTRAC-U6-gB2m-CAR19 and Lv-U6-gTRAC-mU6-gB2m-CAR19 T cells. Absolute number of gRNA was normalized to 10^6 Gapdh mRNA.

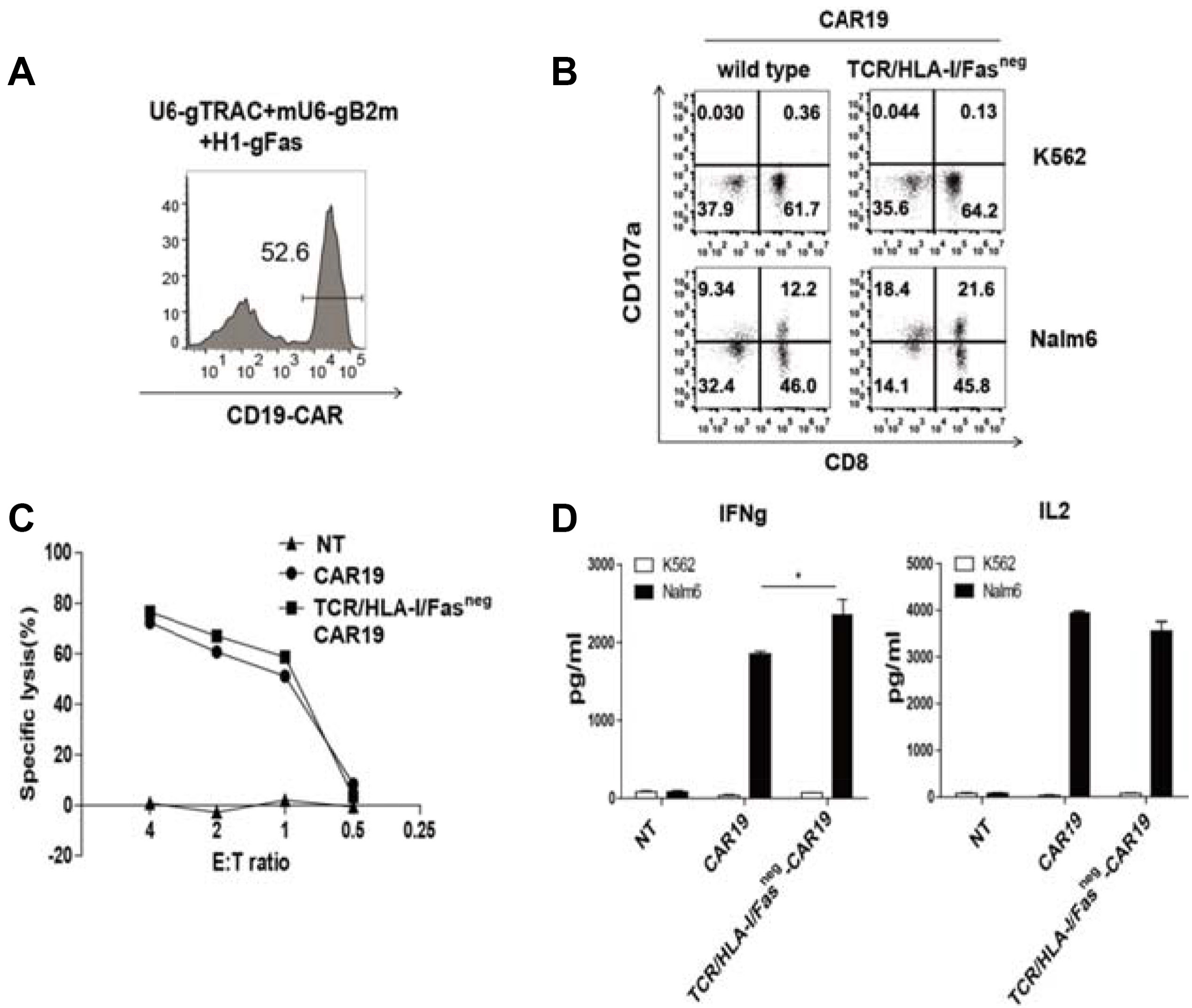

Supplementary Figure 4: Enhanced function of Fas ablated universal CAR T cells. (**A**) Expression of CAR19 on T cells re-directed with one-shot Lv-U6-gTRAC-mU6-gB2m- H1-gFas-CAR19 lentivirus as determined by flow cytometry. Function of TCR/HLA-I/Fas triple negative CAR19 T cells was tested by (**B**) CD107a release assay, (**C**) killing assay and (**D**) cytokine secretion.

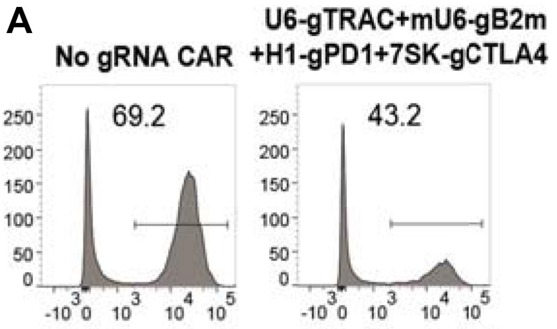

Supplementary Figure 5: Expression of PSCA-CAR in quadruple gene ablated CAR T cells. (**A**) Expression of PSCA-CAR on T cells re-directed with one-shot Lv-U6-gTRAC-mU6-gB2m- H1-gPD1-7SK-gCTLA4-PSCA-CAR lentivirus as determined by flow cytometry
